# A Novel Ferroptosis-Related Pathway for Regulating Immune Checkpoints in Clear Cell Renal Cell Carcinoma

**DOI:** 10.3389/fonc.2021.678694

**Published:** 2021-07-21

**Authors:** Su Gao, Hailong Ruan, Jingchong Liu, Yuenan Liu, Di Liu, Junwei Tong, Jian Shi, Hongmei Yang, Tianbo Xu, Xiaoping Zhang

**Affiliations:** ^1^ Department of Geriatrics, Union Hospital, Tongji Medical College, Huazhong University of Science and Technology, Wuhan, China; ^2^ Institute of Gerontology, Union Hospital, Tongji Medical College, Huazhong University of Science and Technology, Wuhan, China; ^3^ Department of Urology, Union Hospital, Tongji Medical College, Huazhong University of Science and Technology, Wuhan, China; ^4^ Department of Pathogenic Biology, School of Basic Medicine, Huazhong University of Science and Technology, Wuhan, China

**Keywords:** clear cell renal cell carcinoma, ferroptosis, TAZ, WNT10B, immune checkpoints, PD-1

## Abstract

Ferroptosis is a novel form of cell death and plays a role in various diseases, especially tumors. It has been reported that ferroptosis is involved in the growth and progression of clear cell renal cell carcinoma (ccRCC); however, the specific molecular mechanisms are still unclear. In this study, we constructed a four-gene signature (FeSig) of ferroptosis-related genes *via* Cox regression analysis. ROC and survival analyses indicated that FeSig had good diagnostic and prognostic value. Further analysis revealed that ferroptosis was associated with tumor immunity in ccRCC. Next, weighted gene co-expression network analysis was performed to identify the potential regulatory mechanisms. Combined with correlation and survival analyses, the TAZ/WNT10B axis was identified as a tumor immune-related regulatory pathway. In conclusion, these findings suggest that ferroptosis is correlated with tumor immunity. The TAZ/WNT10B axis may be a novel biomarker and therapeutic target for immunotherapy in ccRCC.

## Introduction

Renal cell carcinoma (RCC) is a common neoplasm that originates from renal tubular epithelial cells and accounts for 2–3% of all malignant tumors in adults (1). In the USA, it is estimated that there will be approximately 70,000 new cases and 14,000 deaths due to this cancer in 2020. The 5-year relative survival is 75.2% (2). Clear cell renal cell carcinoma (ccRCC) is the most common histological subtype and accounts for over 70% of RCCs (1). Due to high resistance to conventional chemoradiotherapy, only limited therapies are currently available (3, 4). At present, immunotherapy, as a novel treatment, is gradually being applied in ccRCC therapy (5). Immune checkpoint inhibitors (ICIs), such as PD-1 and CTLA-4 inhibitors (6, 7), have improved clinical responses and quality of life for some patients. However, some patients are insensitive to ICIs (8). Therefore, it is necessary to further study the regulatory mechanisms of immune checkpoints in ccRCC.

Ferroptosis is a novel form of cell death caused by iron-dependent oxidative damage (9). Due to the failure of glutathione peroxidase (GPX4), a large number of reactive oxygen radicals (ROS) accumulate on membrane lipids (10). In recent years, many studies have verified that ferroptosis plays a vital role in degenerative diseases (*i.e.*, Alzheimer’s and Parkinson’s diseases) (11, 12), ischemia–reperfusion injury (13), and tumors (14). Zhang et al. showed that BAP1 inhibits tumor progression by promoting cellular ferroptosis (15). Furthermore, it has been reported that ferroptosis is closely associated with the therapeutic effect of immunotherapy. Wang et al. verified that CD8+ T cells enhanced the effect of immunotherapy by promoting ferroptosis of tumor cells (16). Interestingly, Lang et al. indicated that immunotherapy sensitizes tumors to radiotherapy by inhibiting SLC7A11 and reducing cystine uptake (17). However, the interaction between ferroptosis and immunotherapy in ccRCC is still unclear. In this study, we constructed a ferroptosis-related gene signature (FeSig) and analyzed the correlation between FeSig and immune checkpoints to identify novel therapeutic targets in ccRCC.

## Methods and Materials

### Data Download and Study Design

**Figure 1:**
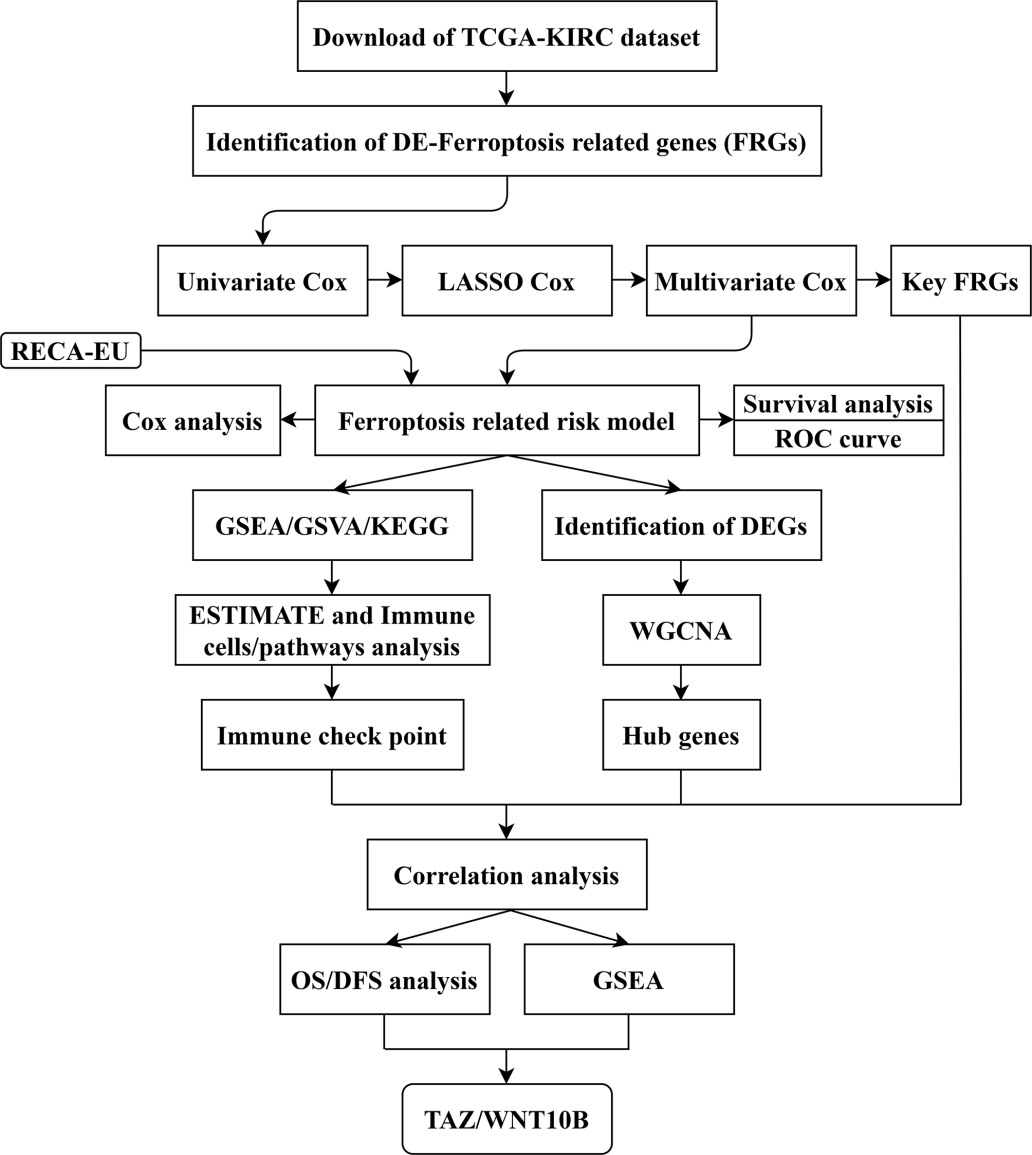
Flow chart of study design. The data collection and study process were shown in a flow chart. TCGA, The Cancer Genome Atlas; KIRC, kidney clear cell carcinoma; FRGs, ferroptosis-related genes; LASSO, least absolute shrinkage and selection operator; ROC, receiver operating characteristic; GSEA, gene set enrichment analysis; GSVA, gene set variation analysis; KEGG: Kyoto Encyclopedia of Genes and Genomes; DEGs, differentially expressed genes; ESTIMATE, Estimation of STromal and Immune cells in Malignant Tumor tissues using Expression data; WGCNA, weighted gene co-expression network analysis; OS, overall survival; DFS, disease-free survival.

The gene expression data and clinical data were obtained from The Cancer Genome Atlas (TCGA-KIRC, https://www.cancer.gov/tcga) and International Cancer Genome Consortium (ICGC-RECA-EU, https://dcc.icgc.org/) databases. An immunotherapy (immune checkpoint inhibitor, ICI) dataset of clear cell renal cell carcinoma (ccRCC) with clinical data and gene expression data was obtained from the study of Miao et al. (18). In addition, the study process is shown in a flow chart (**Figure 1**).

#### Identification of Differentially Expressed Genes and Ferroptosis-Related Genes

The “DESeq2” (19) and “edgeR“ (20) packages were used to screen DEGs with p-value <0.05 and |log2FC| > 1.5. The Wilcoxon test was performed to identify DE-FRGs with a p-value <0.05 and |log2FC| >1.

#### Protein–Protein Interaction Network Analysis

The DE-FRGs were inputted into the STRING online tool (https://string-db.org) to construct the PPI network. Then, Cytoscape software (21) was used to analyze the PPI network.

### Cox Regression Analysis and Construction of a Proportional Hazards Model

The “survival” package (https://CRAN.R-project.org/package=survival) was utilized to perform univariate and multivariate Cox regression analyses. The “glmnet” (22) and “survival” packages were used to perform least absolute shrinkage and selection operator (LASSO) regression analysis. Through integrated Cox analysis, four key FRGs were screened to construct the risk model (FeSig). The risk score (RS) formula was as follows:

$$\text{RS}=\sum_{i=1}^{\text{n}}Coef(i)X(i)$$

Where *Coef* (*i*) represents the coefficient, and *X*(*i*) represents the expression of selected genes.

### Principal Component Analysis and T-Distributed Stochastic Neighbor Embedding Analysis

The “stats” and “limma” packages (23) were used to perform PCA. The “Rtsne” package (https://CRAN.R-project.org/package=Rtsne) was utilized to perform t-SNE analysis. PCA and t-SNE analysis were both used to explore the distribution of different groups.

### Survival Analysis and Receiver Operator Characteristic Curve Analysis

According to the median gene expression/risk score, ccRCC patients were divided into a high group and a low group. Then, survival curves of overall survival (OS) and disease-free survival (DFS) were drawn by the “survival” package in R and GraphPad software (version 7.0). A p-value <0.05 was considered statistically significant. Moreover, the “survivalROC” (https://CRAN.R-project.org/package=survivalROC) package was used to generate a time-dependent ROC curve to evaluate the predictive value of the risk model.

### Gene Set Enrichment Analysis and Gene Set Variation Analysis

All patients were divided into two groups (high group and low group) according to the median gene expression/risk score. GSEA (24) was performed to discover potential mechanisms and downstream signaling pathways. Moreover, the “GSVA” package (25) was utilized to find differential signaling pathways between the high group and the low group.

### Kyoto Encyclopedia of Genes and Genomes Pathway Enrichment Analysis

The “clusterProfiler” package (26) was used to conduct KEGG enrichment analysis of DEGs. The results were visualized by the “ggplot2” package (https://CRAN.R-project.org/package=ggplot2) in the R programme. A p-value <0.05 was selected as the cut-off point.

### Weighted Gene Co-Expression Network Analysis

The “WGCNA” package (27) was applied to construct the weighted gene co-expression network of DEGs. First, the TCGA-KIRC cohort was evaluated *via* sample clustering to detect outliers with a height cut-off point = 60. Then, Pearson’s correlation was calculated between each of the gene pairs. Second, a matrix of adjacencies was constructed according to Pearson’s correlation. Then, the adjacencies achieved scale-free topology based on the soft threshold power *β* (**Figure 7A**). Third, the adjacencies were transformed into a topological overlap matrix (TOM). Genes with a high absolute correlation were clustered into the same module. Finally, combined with clinical traits, we calculated the correlation between the module eigengenes (MEs) and clinical traits to screen the clinically significant modules. After that, the correlation between genes and clinical traits (cor.geneTraitSignificance) and the correlation between genes and MEs (cor.geneModuleMembership) were conducted to identify hub genes. In this study, cor.geneTraitSignificance >0.2 and cor.geneModuleMembership >0.6 were selected as the cut-off criteria.

### Human Clinical Specimens

A total of 19 pairs of ccRCC samples and adjacent normal samples (4 cm away from the margin of the tumor tissues) were obtained from Wuhan Union Hospital between 2018 and 2020. The study was approved by the Human Research Ethics Committee of Huazhong University of Science and Technology (HUST), and all patients signed the informed consent.

### RNA Extraction and qRT-PCR

Total RNA was extracted from ccRCC samples and adjacent normal samples using Trizol Reagent (Sigma, USA). Then, cDNA was synthesized using qPCR RT Kit (Vazyme, China). After that, quantitative real time PCR (qRT-PCR) was performed to amplify cDNA.

Primer sequences were listed as follows:

GAPDH Forward: 5′-GCACCGTCAAGGCTGAGAAC-3′;
GAPDH Reverse: 5′-TGGTGAAGACGCCAGTGGA-3′;
TAZ Forward: 5′-CACCGTGTCCAATCACCAGTC-3′;
TAZ Reverse: 5′-TCCAACGCATCAACTTCAGGT-3′;
WNT10B Forward: 5′-CATCCAGGCACGAATGCGA-3′;
WNT10B Reverse: 5′-CGGTTGTGGGTATCAATGAAGA-3′.

### Statistical Analysis

In this study, all data are presented as the mean ± SD. SPSS (version 22.0) and GraphPad Prism (version 7.0) were used to analyze the data. Student’s t-test, Mann–Whitney test, and Pearson’s χ^2^ test were used to conduct statistical analyses. A p-value <0.05 was considered statistically significant.

## Results

### Identification of DE-FRGs and Construction of a Proportional Hazards Model

**Figure 2:**
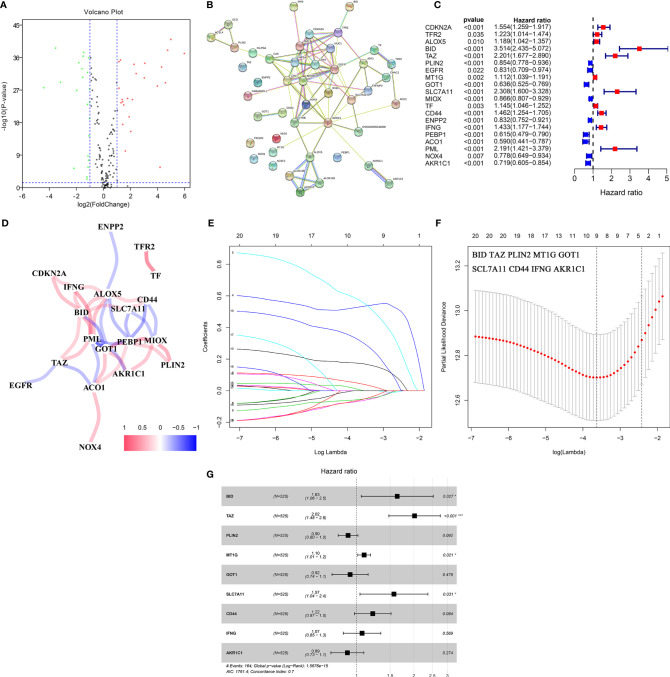
Identification of key DE-FRGs *via* Cox regression analysis. **(A)** The volcano plot of FRGs. **(B)** The protein–protein interaction between FRGs. **(C)** Univariate cox regression analysis revealed that a total of twenty DE-FRGs had prognostic value. **(D)** The correlation network of DE-FRGs. **(E, F)** Through LASSO Cox regression, nine genes were selected as key DE-FRGs for further analysis. **(G)** Four genes (BID, TAZ, MT1G, and SCL7A11) were identified as independent prognostic factors *via* multivariate Cox regression analysis. DE-FRGs, differentially expressed ferroptosis-related genes; LASSO, least absolute shrinkage and selection operator. ***P < 0.001; *P < 0.05.

The gene expression data were obtained from the TCGA-KIRC cohort. According to the cut-off criteria, 46 DE-FRGs were identified (**Figure 2A**). The protein–protein interactions of DE-FRGs were shown in **Figure 2B**. Further analysis indicated that there were 20 DE-FRGs with good prognostic value (**Figure 2C**). The correlation was close and high between these DE-FRGs (**Figure 2D**). Then, LASSO and multivariate Cox regression analyses were applied to construct a prognostic model. A four-gene signature (FeSig) was identified (**Figures 2E–G**). The risk score = 0.49*expression of BID + 0.70*expression of TAZ + 0.09*expression of MT1G + 0.45*expression of SLC7A11.

### The Diagnostic and Prognostic Value of the Four-Gene Signature

**Figure 3:**
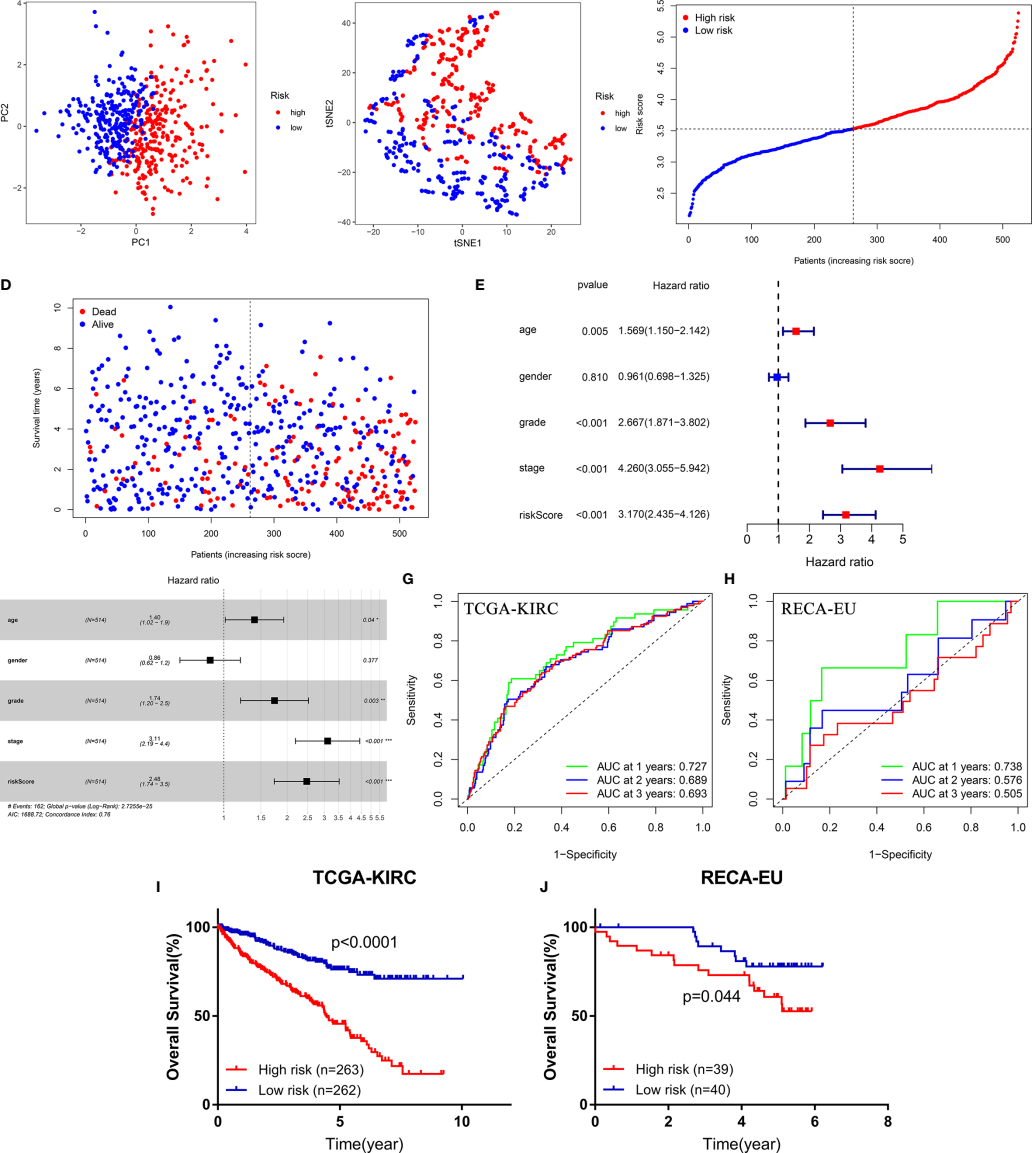
The diagnostic and prognostic value of the four-gene signature (FeSig). **(A, B)** PCA and t-SNE analysis both verified that FeSig could significantly divide patients into different risk groups. **(C)** The distribution and median value of the risk scores in the TCGA cohort. **(D)** Patients with high-risk score had poor OS. **(E, F)** Univariate and multivariate Cox regression analyses indicated that risk score was an independent prognostic factor (HR = 2.48, p < 0.001). **(G, H)** The risk score had good diagnostic value. **(I, J)** High-risk group predicted poor OS both in TCGA and ICGC cohort. FeSig, ferroptosis-related gene signature; PCA, principal component analysis; t-SNE, t-distributed stochastic neighbor embedding; TCGA, The Cancer Genome Atlas; OS, overall survival; ICGC, International Cancer Genome Consortium.

PCA and t-SNE analysis proved that FeSig could significantly divide patients into different risk groups (**Figures 3A, B**). In the TCGA-KIRC cohort, patients were divided into two groups (high-risk group and low-risk group) according to the median risk score (**Figure 3C**). As shown in **Figure 3D**, patients with a high-risk score had a higher probability of death earlier than those with a low risk score. Further Cox regression analysis revealed that the risk score was an independent prognostic factor (**Figures 3E, F**). Time-dependent ROC curves indicated that the risk score had good predictive performance in both the TCGA-KIRC (**Figure 3G**) cohort and the ICGC-RECA-EU (**Figure 3H**) cohort. Moreover, survival analysis also uncovered that the high-risk group predicted poor OS (**Figures 3I, J**). These findings suggested that FeSig had good diagnostic and prognostic value.

### FeSig Is Closely Correlated With Immune-Related Pathways

**Figure 4:**
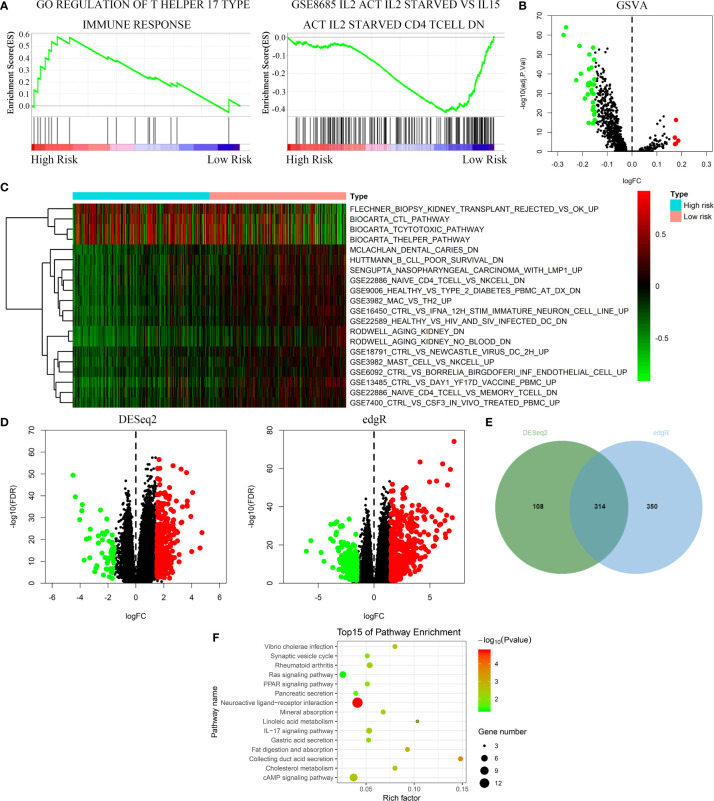
The FeSig was obviously closely associated with immune-related pathways. **(A–C)** GSEA and GSVA showed that FeSig was correlated to immune cell related pathways. **(D, E)** A total of 314 DEGs were identified *via* DESeq2 and edgR package. **(F)** The DEGs were mainly enriched in immune-related signaling pathways. FeSig, ferroptosis-related gene signature; GSEA, gene set enrichment analysis; GSVA, gene set variation analysis; DEGs, differentially expressed genes.

**Figure 5:**
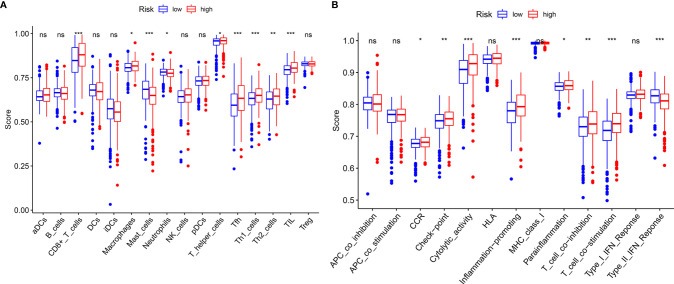
ssGSEA analysis of FeSig. **(A, B)** The ssGSEA score of 16 immune cells and 13 immune-related functions between high-risk group and low-risk group. ssGSEA, single sample gene set enrichment analysis. ***P < 0.001; **P < 0.01; *P < 0.05; ns, no significance.

GSEA, GSVA, and KEGG analyses were performed to identify potential downstream signaling pathways of FeSig. As shown in **Figure 4A**, FeSig was associated with immune cell-related pathways. Analogously, GSVA also verified that there were many differentially enriched immune cell-related pathways between the high-risk group and the low-risk group (**Figures 4B, C**). Then, 314 DEGs were identified *via* DESeq2 and edgeR package in the R programme (**Figures 4D, E**). Further KEGG pathway enrichment analysis indicated that these DEGs were mainly enriched in immune-related pathways (**Figure 4F**, Ras signaling pathway, PPAR signaling pathway, and IL-17 signaling pathway). To further study the correlation between FeSig and immune status, we quantified the enrichment scores of diverse immune cell subpopulations, related functions, or pathways with ssGSEA. As shown in **Figure 5A**, nine immune cell subpopulations (CD8+ T cells, macrophages, mast cells, neutrophils, T helper cells, Tfhs, Th1 cells, Th2 cells, and TILs) were clearly different between the high-risk group and the low-risk group. For immune-related functions, seven pathways (CCR, checkpoint, cytolytic activity, inflammation promotion, parainflammation, T cell coinhibition, and T cell costimulation) had higher scores in the high-risk group, and only the type II IFN response had higher scores in the low-risk group (**Figure 5B**). These findings revealed that FeSig is significantly associated with the regulation of tumor immunity.

### Potential of the FeSig as an Indicator of Response to Anti-PD-1 Therapy

**Figure 6:**
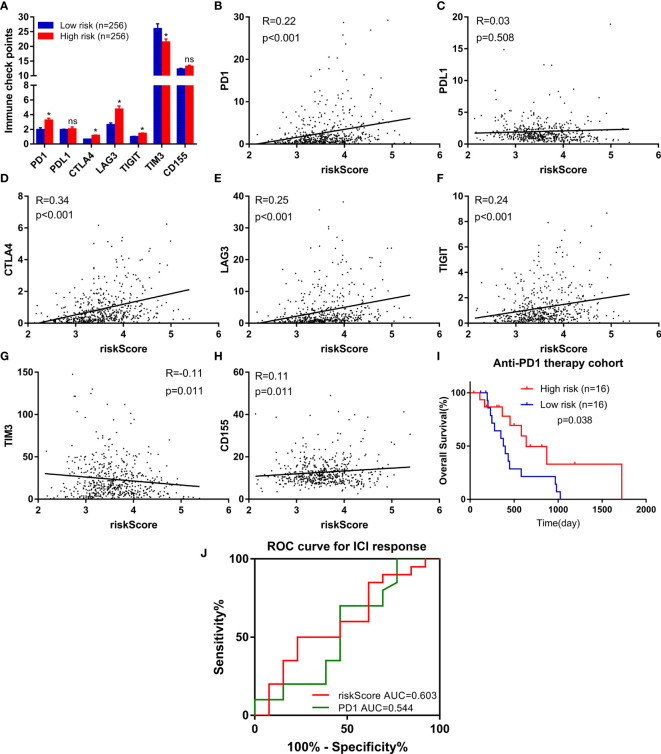
FeSig was a potential indicator of response to anti-PD-1 therapy. **(A)** The expression level of immune checkpoints between high-risk group and low-risk group. **(B–H)** The correlation between immune checkpoints and risk score. **(I)** High risk group of patients with anti-PD-1 monotherapy had better OS. **(J)** ROC curve of ICI response showed that FeSig had good diagnostic value. OS, overall survival; ROC, receiver operating characteristic; ICI, immune checkpoint inhibitor. *P < 0.05; ns, no significance.

Due to the wide popularization of immune checkpoint inhibitors in ccRCC, we further focused on the correlation between FeSig and immune checkpoint pathways. As shown in **Figure 6A**, we found that PD-1, CTLA4, LAG3, and TIGIT were upregulated in the high-risk group and TIM-3 was downregulated in the high-risk group. Moreover, correlation analysis indicated that the riskScore was significantly positively correlated with PD-1, CTLA4, LAG3, and TIGIT (**Figures 6B–H**). These findings suggested that FeSig may be a biomarker of the response to immunotherapy. Therefore, the predictive value of FeSig was tested in an immunotherapy dataset of ccRCC. Survival analysis verified that there was a significant difference (log-rank p = 0.038) between the high- and low-risk groups of patients with anti-PD-1 monotherapy. Patients in the high-risk group had longer OS than those in the low-risk group (**Figure 6I**). The ROC curve of ICI response (**Figure 6J**) revealed that the diagnostic value of FeSig (AUC = 0.603) was better than that of PD-1 (AUC = 0.544).

### Identification of the TAZ/WNT10B Axis as an Immune Checkpoint Regulatory Pathway in ccRCC

**Figure 7:**
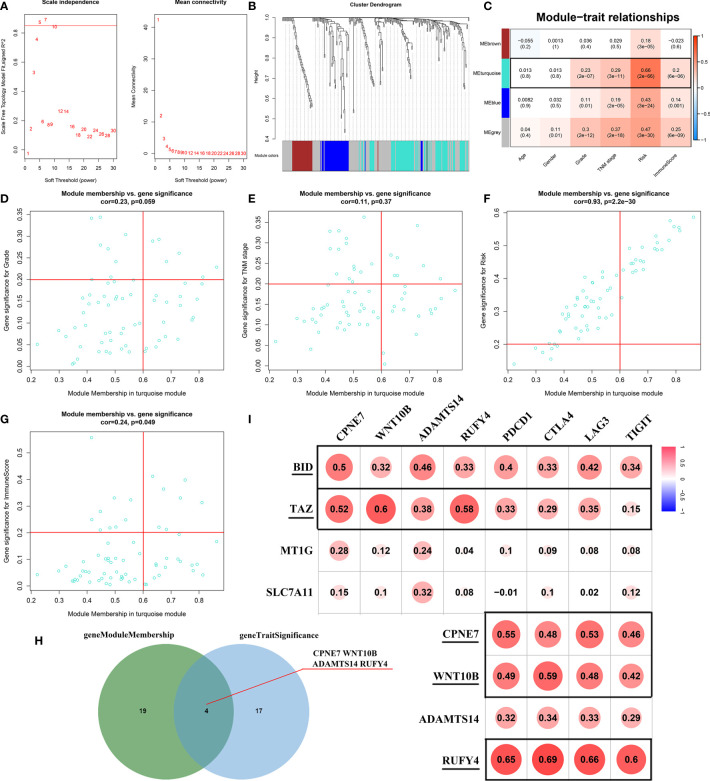
Identification of key genes of regulating immune checkpoints. **(A)** Analysis of scale-free fit parameter and mean connectivity for various soft-thresholding power. **(B)** Dendrogram of DEGs clustered based on a dissimilarity measure (1-TOM). The DEGs were divided into four modules (brown, turquoise, blue, and gray). **(C)** The correlation between clinical traits and modules. MEturquoise was selected as the hub module. **(D–H)** Four genes (CPNE7, WNT10B, ADAMTS14, and RUFY4) were regarded as downstream hub genes. **(I)** The correlation among the FeSig (BID, TAZ, MT1G, and SLC7A11), downstream hub genes (CPNE7, WNT10B, ADAMTS14, and RUFY4), and immune checkpoints (PD-1, CTLA4, LAG3, and TIGIT). BID, TAZ, CPNE7, WNT10B, and RUFY4 were selected for further analysis. DEGs, differentially expressed genes; TOM, topological overlap matrix.

**Figure 8:**
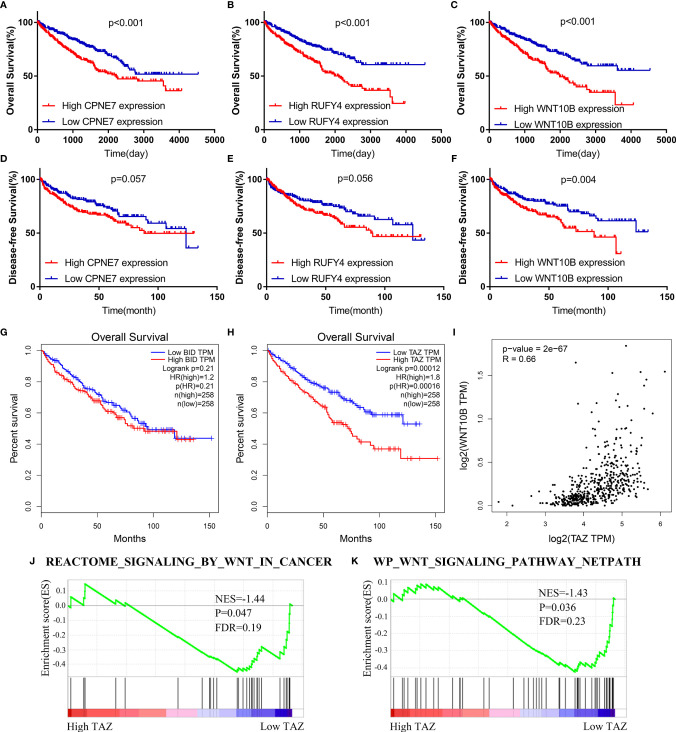
TAZ/WNT10B axis was identified as a potential immune checkpoint regulatory pathway. **(A–F)** WNT10B had better prognostic value than CPNE7 and RUFY4 according to survival analysis of OS and DFS. **(G, H)** Survival analysis revealed that TAZ had better prognostic value than BID. **(I)** TAZ was closely positively correlated to WNT10B (R = 0.66). **(J, K)** Low TAZ group was enriched in WNT signaling pathways. OS, overall survival; DFS, disease-free survival.

The WGCNA algorithm was performed to find further downstream targets of four-gene signature (FeSig). As shown in **Figure 7A**, *β* = 5 was selected as the soft threshold power in this study. The DEGs between high-risk group and low-risk group were divided into four modules according to the expression level of each gene (**Figure 7B**). Combined with the clinical information, we found that the MEturquoise module was significantly positively associated with grade (R = 0.23, p = 2e-07), TNM stage (R = 0.29, p = 3e-11), risk (R = 0.66, p = 2e-66), and ImmuneScore (R = 0.2, p = 6e-06) (**Figure 7C**). According to cor.geneTraitSignificance >0.2 and cor.geneModuleMembership >0.6, CPNE7, WNT10B, ADAMTS14, and RUFY4 were regarded as downstream hub genes of FesSig (**Table 1**, **Figures 7D–H**). Moreover, we analyzed the correlation among four-gene signature (FeSig) (BID, TAZ, MT1G, and SLC7A11), downstream hub genes (CPNE7, WNT10B, ADAMTS14, and RUFY4), and immune checkpoints (PD-1, CTLA4, LAG3, and TIGIT) to further discover potential molecular mechanisms of tumor immunity. According to the correlation value, BID, TAZ, CPNE7, WNT10B, and RUFY4 were selected for subsequent analysis (**Figure 7I**). Survival analyses of OS and DFS proved that WNT10B had better prognostic prediction performance than CPNE7 and RUFY4 (**Figures 8A–F**). Similarly, TAZ had better prognostic value than BID (**Figures 8G, H**). Therefore, TAZ and WNT10B were selected for further study. In addition, the expression of TAZ was clearly positively correlated with WNT10B (**Figure 8I**, R = 0.66, p = 2e-67). Interestingly, GSEA verified that the low TAZ group was enriched in WNT signaling pathway (**Figures 8J, K**). These findings indicated that TAZ might regulate tumor immunity through mediating WNT10B (WNT signaling pathway) in ccRCC.

**Table 1:** Hub genes were identified *via* WGCNA.

Gene	cor.geneModuleMembership	cor.geneTraitSignificance			
	Turquoise	Grade	TNM	Risk	ImmuneScore
CPNE7	0.63	0.29	0.27	0.49	0.41
WNT10B	0.81	0.21	0.21	0.55	0.31
ADAMTS14	0.66	0.29	0.26	0.46	0.22
RUFY4	0.75	0.20	0.24	0.49	0.40

WGCNA, weighted gene co-expression network analysis.

### TAZ/WNT10B Was Closely Correlated With TNM/Grade Stage and UpRegulated in ccRCC Tissue

**Figure 9:**
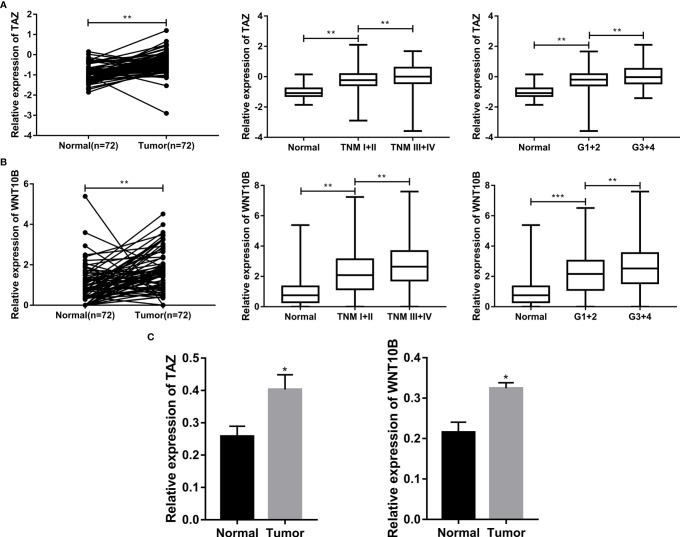
TAZ/WNT10B was closely correlated with TNM/Grade stage and up-regulated in ccRCC tissue. **(A)** In TCGA-KIRC dataset, TAZ was elevated in ccRCC and positively correlated with TNM/Grade stage. **(B)** In TCGA-KIRC dataset, WNT10B was elevated in ccRCC and positively correlated with TNM/Grade stage. **(C)** The mRNA expression level of TAZ/WNT10B was upregulated in ccRCC tissues. ccRCC, clear cell renal cell carcinoma. ***P < 0.001; **P < 0.01; *P < 0.05.

We further analyzed the correlation between the expression level of TAZ/WNT10B and TNM/Grade stage and verified their mRNA expression level in ccRCC tissues. As shown in **Figures 9A, B**, TAZ and WNT10B were both elevated and positively correlated with TNM/Grade stage in ccRCC. Moreover, qRT-PCR assay also indicated that TAZ and WNT10B were upregulated in ccRCC tissues (**Figure 9C**).

## Discussion

Recently, immunotherapy has emerged as a promising approach for cancer treatment, which mainly includes non-specific immunostimulation, immune checkpoint inhibitors (ICIs), tumor vaccines, and adoptive cellular immunotherapy (28). In metastatic RCC (mRCC), immune checkpoint inhibitors have changed the treatment paradigm because most patients with newly diagnosed mRCC are now treated with these medicines. It has been reported that immune checkpoint inhibitors have provided significant clinical benefit for mRCC patients in multiple clinical trials (29). However, there are still some patients without good effects due to adverse reactions and drug resistance to ICIs (30). Therefore, it is necessary to study the molecular mechanisms of drug resistance and reduce the adverse reactions to ICIs. At present, some studies have reported that the induction of ferroptosis enhances the effect of ICIs (16). Interestingly, many ferroptosis-related genes are abnormally expressed in ccRCC and might be potential therapeutic targets. However, the correlation between ferroptosis and immune checkpoints in ccRCC is still unclear.

In this study, a ferroptosis-related risk model (FeSig) was constructed through Cox regression analysis. We found that FeSig had good diagnostic/prognostic value and was closely associated with immune checkpoints. Moreover, patients with high-risk scores had better OS than those with low-risk scores after PD-1 inhibitor treatment, which indicated that FeSig was a potential prognostic biomarker for immunotherapy. Then, the TAZ/WNT10B axis was identified as a potential regulatory pathway of immune checkpoints *via* WGCNA and correlation analysis.

TAZ (Tafazzin) is a transcriptional regulator and plays a vital role in tumorigenesis and tumor progression of most solid tumors (31). TAZ stimulates cell proliferation by regulating DNA duplication and mitosis (32). It has been reported that TAZ maintains plasticity in cell–ECM adhesion and favors cytoskeletal remodeling to promote tumor metastasis (33). TAZ is also regarded as an important regulator of ferroptosis. Two research groups recently reported that TAZ promoted tumor cell ferroptosis by regulating members of the NOX family (34, 35). Furthermore, many studies have verified that TAZ is closely involved in tumor immunity and the microenvironment. TAZ mediates the expression of tumor-secreted factors to drive the differentiation and recruitment of immune suppressive cells, such as tumor-associated macrophages (TAMs) (36) and regulatory T cells (Tregs) (37). In addition, TAZ promotes immune evasion of tumor cells by regulating the expression of immune checkpoints. Feng et al. indicated that tumor cell-derived lactate activated the TAZ/PD-L1 axis to enhance tumor evasion from the immune response (38). Similarly, a recent study also reported that the TAZ/YAP/TEAD signaling pathway increased PD-L1 promoter activity and induced immune evasion of tumors (39). In our study, we found that the expression of TAZ was highly positively associated with PD-1, which suggested that TAZ was a potential therapeutic target of immunotherapy in ccRCC.

To study the specific molecular mechanisms of TAZ, WGCNA and GSEA were performed and indicated that WNT10B is a potential downstream target of TAZ. WNT10B (Wnt Family Member 10B) is a regulator encoding secreted proteins and activating the Wnt signaling cascade (40). It has been reported that the Wnt signaling pathway is closely involved in regulating immune checkpoints. Notably, the expression of PD-L1 has been proven to be regulated by MYC, which is a well-documented target of the Wnt signaling pathway (41). Moreover, inhibiting the Wnt/*β*-catenin axis can promote antitumor immunity by suppressing PD-L1 expression (42). In this study, we also found that TAZ was significantly positively correlated with WNT10B and that high WNT10B predicted poor OS/DFS in ccRCC. These findings suggested that the TAZ/WNT10B axis might regulate tumor immunity by activating the WNT signaling pathway.

In conclusion, the TAZ/WNT10B axis (ferroptosis-related pathway) is regarded as a tumor immune-related regulatory pathway *via* integrated bioinformatics analysis. Further analysis revealed that immune checkpoints are potential targets of the TAZ/WNT10B pathway. Therefore, the TAZ/WNT10B axis is expected to be a novel therapeutic target of immunotherapy in ccRCC. However, the specific mechanisms still require further research.

## Data Availability Statement

Publicly available datasets were analyzed in this study. This data can be found here: TCGA-KIRC, https://www.cancer.gov/tcga and ICGC-RECA-EU, https://dcc.icgc.org https://science.sciencemag.org/content/suppl/2018/01/03/science.aan5951.DC1.

## Ethics Statement

The studies involving human participants were reviewed and approved by Human Research Ethics Committee of Huazhong University of Science and Technology (HUST). The patients/participants provided their written informed consent to participate in this study. Written informed consent was obtained from the individual(s) for the publication of any potentially identifiable images or data included in this article.

## Author Contributions

XZ designed this study. TX and SG performed data collection and analysis. TX, SG, HR, and JL performed the majority of the experiments. TX and SG wrote the manuscript and contributed to preparing and making figures and tables. YL, DL, and JT collected the clinical samples and managed the clinical data. JT and JS reviewed the relevant literature. HY and XZ provided conceptual advice and critically reviewed the manuscript. All authors contributed to the article and approved the submitted version.

## Funding

This study was supported by the Key Research and Development Plan in China (grant no. 2017YFB1303100), the National Natural Science Foundation of China (grant nos. 81672524, 81672528, and 81874090), the Hubei Provincial Natural Science Foundation of China (grant no. 2018CFA038), the Independent Innovation Foundation of Huazhong University of Science and Technology (grant no. 118530309), and the Clinical Research Physician Program of Tongji Medical College, Huazhong University of Science and Technology (grant no. 5001530015).

## Conflict of Interest

The authors declare that the research was conducted in the absence of any commercial or financial relationships that could be construed as a potential conflict of interest.

## Abbreviations

ccRCC, clear cell renal cell carcinoma; RCC, renal cell carcinoma; ICIs, immune checkpoint inhibitors; ROS, reactive oxygen radicals; FeSig, ferroptosis-related gene signature; TCGA, The Cancer Genome Atlas; ICGC, International Cancer Genome Consortium; DEGs, differentially expressed genes; DE-FRGs, differentially ferroptosis-related genes; PPI, protein–protein interaction; LASSO, least absolute shrinkage and selection operator; RS: risk score; PCA: principal component analysis; t-SNE: t-distributed stochastic neighbor embedding; ROC, receiver operator characteristic; GSEA, gene set enrichment analysis; GSVA, gene set variation analysis; KEGG, Kyoto Encyclopedia of Genes and Genomes; WGCNA, weighted gene co-expression network analysis; TOM, topological overlap matrix; MEs, module eigengenes; OS, overall survival; AUC, area under the curve; DFS, disease-free survival; TAM, tumor associated macrophages.

## References

[1] Moch H Cubilla AL Humphrey PA Reuter VE Ulbright TM. The 2016 WHO Classification of Tumours of the Urinary System and Male Genital Organs-Part A: Renal, Penile, and Testicular Tumours. Eur Urol (2016) 70:93–105. 10.1016/j.eururo.2016.02.029 26935559

[2] Siegel RL Miller KD Jemal A. Cancer Statistics, 2020. CA Cancer J Clin (2020) 70:7–30. 10.3322/caac.21590 31912902

[3] Campbell S Uzzo RG Allaf ME Bass EB Cadeddu JA Chang A. Renal Mass and Localized Renal Cancer: AUA Guideline. J Urol (2017) 198:520–9. 10.1016/j.juro.2017.04.100 28479239

[4] Ljungberg B Bensalah K Canfield S Dabestani S Hofmann F Hora M. EAU Guidelines on Renal Cell Carcinoma: 2014 Update. Eur Urol (2015) 67:913–24. 10.1016/j.eururo.2015.01.005 25616710

[5] Deleuze A Saout J Dugay F Peyronnet B Mathieu R Verhoest G. Immunotherapy in Renal Cell Carcinoma: The Future Is Now. Int J Mol Sci (2020) 21:2532. 10.3390/ijms21072532 PMC7177761 32260578

[6] Carosella ED Ploussard G LeMaoult J Desgrandchamps F. A Systematic Review of Immunotherapy in Urologic Cancer: Evolving Roles for Targeting of CTLA-4, PD-1/PD-L1, and HLA-G. Eur Urol (2015) 68:267–79. 10.1016/j.eururo.2015.02.032 25824720

[7] Rotte A. Combination of CTLA-4 and PD-1 Blockers for Treatment of Cancer. J Exp Clin Cancer Res (2019) 38:255. 10.1186/s13046-019-1259-z 31196207 PMC6567914

[8] Fares CM Van Allen EM Drake CG Allison JP Hu-Lieskovan S. Mechanisms of Resistance to Immune Checkpoint Blockade: Why Does Checkpoint Inhibitor Immunotherapy Not Work for All Patients? Am Soc Clin Oncol Educ Book (2019) 39:147–64. 10.1200/edbk_240837 31099674

[9] Dixon SJ Lemberg KM Lamprecht MR Skouta R Zaitsev EM Gleason CE. Ferroptosis: An Iron-Dependent Form of Nonapoptotic Cell Death. Cell (2012) 149:1060–72. 10.1016/j.cell.2012.03.042 PMC3367386 22632970

[10] Stockwell BR Friedmann Angeli JP Bayir H Bush AI Conrad M Dixon SJ. Ferroptosis: A Regulated Cell Death Nexus Linking Metabolism, Redox Biology, and Disease. Cell (2017) 171:273–85. 10.1016/j.cell.2017.09.021 PMC5685180 28985560

[11] Yan N Zhang J. Iron Metabolism, Ferroptosis, and the Links With Alzheimer’s Disease. Front Neurosci (2019) 13:1443. 10.3389/fnins.2019.01443 32063824 PMC7000453

[12] Guiney SJ Adlard PA Bush AI Finkelstein DI Ayton S. Ferroptosis and Cell Death Mechanisms in Parkinson’s Disease. Neurochem Int (2017) 104:34–48. 10.1016/j.neuint.2017.01.004 28082232

[13] Li Y Feng D Wang Z Zhao Y Sun R Tian D. Ischemia-Induced ACSL4 Activation Contributes to Ferroptosis-Mediated Tissue Injury in Intestinal Ischemia/Reperfusion. Cell Death Differ (2019) 26:2284–99. 10.1038/s41418-019-0299-4 PMC6889315 30737476

[14] Hassannia B Vandenabeele P Vanden Berghe T. Targeting Ferroptosis to Iron Out Cancer. Cancer Cell (2019) 35:830–49. 10.1016/j.ccell.2019.04.002 31105042

[15] Zhang Y Shi J Liu X Feng L Gong Z Koppula P. BAP1 Links Metabolic Regulation of Ferroptosis to Tumour Suppression. Nat Cell Biol (2018) 20:1181–92. 10.1038/s41556-018-0178-0 PMC6170713 30202049

[16] Wang W Green M Choi JE Gijón M Kennedy PD Johnson JK. CD8(+) T Cells Regulate Tumour Ferroptosis During Cancer Immunotherapy. Nature (2019) 569:270–4. 10.1038/s41586-019-1170-y PMC6533917 31043744

[17] Lang X Green MD Wang W Yu J Choi JE Jiang L. Radiotherapy and Immunotherapy Promote Tumoral Lipid Oxidation and Ferroptosis via Synergistic Repression of SLC7A11. Cancer Discov (2019) 9:1673–85. 10.1158/2159-8290.cd-19-0338 PMC6891128 31554642

[18] Miao D Margolis CA Gao W Voss MH Li W Martini DJ. Genomic Correlates of Response to Immune Checkpoint Therapies in Clear Cell Renal Cell Carcinoma. Science (2018) 359:801–6. 10.1126/science.aan5951 PMC6035749 29301960

[19] Love MI Huber W Anders S. Moderated Estimation of Fold Change and Dispersion for RNA-Seq Data With Deseq2. Genome Biol (2014) 15:550. 10.1186/s13059-014-0550-8 25516281 PMC4302049

[20] Robinson MD McCarthy DJ Smyth GK. Edger: A Bioconductor Package for Differential Expression Analysis of Digital Gene Expression Data. Bioinformatics (2009) 26:139–40. 10.1093/bioinformatics/btp616 PMC2796818 19910308

[21] Shannon P Markiel A Ozier O Baliga NS Wang JT Ramage D. Cytoscape: A Software Environment for Integrated Models of Biomolecular Interaction Networks. Genome Res (2003) 13:2498–504. 10.1101/gr.1239303 PMC403769 14597658

[22] Friedman J Hastie T Tibshirani R. Regularization Paths for Generalized Linear Models *via* Coordinate Descent. J Stat Softw (2010) 33:1–22. 10.18637/jss.v033.i01 20808728 PMC2929880

[23] Ritchie ME Phipson B Wu D Hu Y Law CW Shi W. Limma Powers Differential Expression Analyses for RNA-Sequencing and Microarray Studies. Nucleic Acids Res (2015) 43:e47. 10.1093/nar/gkv007 25605792 PMC4402510

[24] Subramanian A Tamayo P Mootha VK Mukherjee S Ebert BL Gillette MA. Gene Set Enrichment Analysis: A Knowledge-Based Approach for Interpreting Genome-Wide Expression Profiles. Proc Natl Acad Sci USA (2005) 102:15545–50. 10.1073/pnas.0506580102 PMC1239896 16199517

[25] Hänzelmann S Castelo R Guinney J. GSVA: Gene Set Variation Analysis for Microarray and RNA-Seq Data. BMC Bioinf (2013) 14:7. 10.1186/1471-2105-14-7 PMC3618321 23323831

[26] Yu G Wang LG Han Y He QY. Clusterprofiler: An R Package for Comparing Biological Themes Among Gene Clusters. Omics (2012) 16:284–7. 10.1089/omi.2011.0118 PMC3339379 22455463

[27] Langfelder P Horvath S. WGCNA: An R Package for Weighted Correlation Network Analysis. BMC Bioinf (2008) 9:559. 10.1186/1471-2105-9-559 PMC2631488 19114008

[28] Inthagard J Edwards J Roseweir AK. Immunotherapy: Enhancing the Efficacy of This Promising Therapeutic in Multiple Cancers. Clin Sci (2019) 133:181–93. 10.1042/cs20181003 30659159

[29] Parikh M Bajwa P. Immune Checkpoint Inhibitors in the Treatment of Renal Cell Carcinoma. Semin Nephrol (2020) 40:76–85. 10.1016/j.semnephrol.2019.12.009 32130969 PMC7164329

[30] Postow MA Sidlow R Hellmann MD. Immune-Related Adverse Events Associated With Immune Checkpoint Blockade. N Engl J Med (2018) 378:158–68. 10.1056/NEJMra1703481 29320654

[31] Zanconato F Cordenonsi M Piccolo S. YAP/TAZ at the Roots of Cancer. Cancer Cell (2016) 29:783–803. 10.1016/j.ccell.2016.05.005 27300434 PMC6186419

[32] Jang W Kim T Koo JS Kim SK Lim DS. Mechanical Cue-Induced YAP Instructs Skp2-Dependent Cell Cycle Exit and Oncogenic Signaling. EMBO J (2017) 36:2510–28. 10.15252/embj.201696089 PMC5579353 28673931

[33] Mason DE Collins JM Dawahare JH Nguyen TD Lin Y Voytik-Harbin SL. YAP and TAZ Limit Cytoskeletal and Focal Adhesion Maturation to Enable Persistent Cell Motility. J Cell Biol (2019) 218:1369–89. 10.1083/jcb.201806065 PMC6446844 30737263

[34] Yang WH Huang Z Wu J Ding CC Murphy SK Chi JT. A TAZ-ANGPTL4-NOX2 Axis Regulates Ferroptotic Cell Death and Chemoresistance in Epithelial Ovarian Cancer. Mol Cancer Res (2020) 18:79–90. 10.1158/1541-7786.Mcr-19-0691 31641008 PMC6942206

[35] Yang WH Ding CC Sun T Rupprecht G Lin CC Hsu D. The Hippo Pathway Effector TAZ Regulates Ferroptosis in Renal Cell Carcinoma. Cell Rep (2019) 28:2501–8.e4. 10.1016/j.celrep.2019.07.107 31484063 PMC10440760

[36] Zhao X Wang X You Y Wen D Feng Z Zhou Y. Nogo-B Fosters HCC Progression by Enhancing Yap/Taz-Mediated Tumor-Associated Macrophages M2 Polarization. Exp Cell Res (2020) 391:111979. 10.1016/j.yexcr.2020.111979 32246992

[37] Geng J Yu S Zhao H Sun X Li X Wang P. The Transcriptional Coactivator TAZ Regulates Reciprocal Differentiation of T(H)17 Cells and T(reg) Cells. Nat Immunol (2017) 18:800–12. 10.1038/ni.3748 28504697

[38] Feng J Yang H Zhang Y Wei H Zhu Z Zhu B. Tumor Cell-Derived Lactate Induces TAZ-Dependent Upregulation of PD-L1 Through GPR81 in Human Lung Cancer Cells. Oncogene (2017) 36:5829–39. 10.1038/onc.2017.188 28604752

[39] Janse van Rensburg HJ Azad T Ling M Hao Y Snetsinger B Khanal P. The Hippo Pathway Component TAZ Promotes Immune Evasion in Human Cancer Through PD-L1. Cancer Res (2018) 78:1457–70. 10.1158/0008-5472.Can-17-3139 29339539

[40] Wend P Wend K Krum SA Miranda-Carboni GA. The Role of WNT10B in Physiology and Disease. Acta Physiol (Oxf) (2012) 204:34–51. 10.1111/j.1748-1716.2011.02296.x 21447090

[41] Casey SC Tong L Li Y Do R Walz S Fitzgerald KN. MYC Regulates the Antitumor Immune Response Through CD47 and PD-L1. Science (2016) 352:227–31. 10.1126/science.aac9935 PMC4940030 26966191

[42] Wong C Chen C Wu Q Liu Y Zheng P. A Critical Role for the Regulated wnt-myc Pathway in Naive T Cell Survival. J Immunol (2015) 194:158–67. 10.4049/jimmunol.1401238 PMC4272883 25429066

